# Monoclonal antibody infusion reaction with bamlanivimab and etesevimab in a 5-year-old male with coronavirus disease 2019: a case report

**DOI:** 10.1186/s13256-023-03779-3

**Published:** 2023-02-24

**Authors:** Rajapillai L. I. Pillai, Caroline Dziel, Stephanie Vogel, Frank Szczerba, Weijen W. Chang, Ingrid Y. Camelo, Armando Paez

**Affiliations:** 1 Department of Neurology, Boston Children’s Hospital, 300 Longwood Ave, Boston, MA 02115 USA; 2 Department of Pediatrics, UMass-Chan Baystate, 759 Chestnut St, Springfield, MA 01199 USA; 3 Department of Pediatrics, Medical College of Georgia, 1120 15th St, Augusta, GA 30912 USA; 4 Department of Medicine, UMass-Chan Baystate, 759 Chestnut St, Springfield, MA 01199 USA

**Keywords:** Bamlanivimab, Etesevimab, Monoclonal antibody, Infusion reaction, Anaphylactoid, Case report

## Abstract

**Background:**

Bamlanivimab and etesevimab had been granted emergency use authorization in children under 12 years who are at risk of progression from mild/moderate coronavirus disease 2019 to severe disease and hospitalization.

**Case report:**

We report on a 5-year-old white male with preexisting conditions, predisposing him to severe disease, who developed hypoxia and flushing 3 minutes into his infusion, thus meeting the criteria for anaphylaxis.

**Conclusions:**

We believe this patient developed either an immunoglobulin E-mediated anaphylactic or a non-immunoglobulin E-mediated anaphylactoid reaction to bamlanivimab and etesevimab, which is an important possibility to consider on administration.

## Background

Coronavirus disease 2019 (COVID-19) has had a profound effect on the management of patients with complex medical conditions [1, 2]. On 9 February 2021, the Food and Drug Administration (FDA) granted emergency use authorization (EUA) to administer bamlanivimab and etesevimab in adults and children, 12 years of age or older, who weigh more than 40 kg, with mild to moderate COVID-19 at risk for severe infection and hospitalization. On 3 December 2021, the FDA extended this EUA to cover children under the age of 12 [3]. Bamlanivimab and etesevimab are anti-spike neutralizing monoclonal antibodies that target separate, but overlapping, domains of the severe acute respiratory syndrome coronavirus 2 (SARS-CoV-2) spike protein, thus reducing viral load and decreasing resistance to single-antibody treatment [4]. Emergency use had been granted, given promising results of the ongoing BLAZE-1 (NCT04427501) and BLAZE-4 (NCT04634409) clinical trials [5, 6]. Our institution had previously given bamlanivimab and etesevimab successfully in adults and children 12 years and older, weighing more than 40 kg, and once in a patient included in the expanded EUA without complication. Here, we present a case where a patient developed acute hypoxia 3 minutes into his infusion.

## Case report

A 5-year-old white male presented to our institution 1 week after testing positive for SARS-CoV-2 by reverse transcription polymerase chain reaction (RT-PCR). His symptoms had begun the same day as testing and consisted of fever, malaise, cough, and congestion. He had received the first dose of the Pfizer-BioNTech BNT162b2 mRNA vaccine 3 days prior to symptom onset. He had a prior history of biliary atresia with Kasai procedure, portal hypertension, splenomegaly, and neutropenia—all these comorbidities made him high risk for progression to severe disease, and therefore eligible for monoclonal antibody treatment. His past medical history was additionally notable for strabismus with surgical repair, as well as frenectomy. His medications were ursodiol and multivitamins. There was no known history of allergy. Family history was unknown as he was adopted. Baseline labs were obtained at an outside hospital as he was transferred specifically for treatment.

A set of vitals prior to infusion showed a temperature of 98.7 °F, heart rate of 125 beats per minute, respiratory rate of 28 breaths per minute, blood pressure of 108/58 mmHg, and oxygen saturation of 100% on room air. Physical examination was remarkable only for marked splenomegaly; he was otherwise comfortable and interactive. A consent document and fact sheet were shared with the family, and they agreed to proceed with the infusion. Bamlanivimab was given at a dose of 350 mg and etesevimab was given at a dose of 700 mg at a total volume of 30 mL as per the guidelines of the EUA for his weight of 20.4 kg [3]. The infusion was set at a rate for a total infusion time of 21 minutes, approximately 1.5 mL per minute. This was slower than the maximal approved rate in the EUA, which allows for a total infusion time of 16 minutes.

At 3 minutes into the infusion, the patient began coughing and gagging, at which point his oxygen saturation abruptly dropped from 99% just prior to the infusion to 70%. There was no audible stridor, though he was noted to have facial pallor and circumoral cyanosis. The infusion was immediately stopped, and he was placed on 6 L of oxygen via nasal cannula, at which point his pallor and circumoral cyanosis resolved. At this point he was noted to have diffuse flushing, edema of the face, arms, and abdomen, and a petechial rash to his neck and upper chest. Oral diphenhydramine 20 mg was administered, as the patient’s intravenous catheter had come out due to struggling when his nasal cannula was placed. His oxygen saturation improved to 98% after 2 minutes on 6 L of oxygen, so he was transitioned to room air; repeat oxygen saturation was 99%. The decision was made not to continue the infusion, and he was observed overnight without any further acute events. He was discharged home the following day. He followed up with his pediatrician at another institution, who made no report of progressive or worsening symptoms.

## Discussion

Bamlanivimab and etesevimab was granted EUA due to their favorable effects observed in preventing illness, with a 70% lower rate of hospital admission and death compared with placebo [7, 8]. While the BLAZE-4 trial primarily focused on adults, one of the treatment arms of BLAZE-1 included children, which helped form the basis for the FDA’s decision. Adverse events were noted in each treatment group of the BLAZE-1 trial, the most common being nausea and diarrhea. Immediate hypersensitivity reactions were noted in only 9 out of 577 patients who had received an infusion, and only 2 of these were from bamlanivimab and etesevimab (the others were with bamlanivimab alone or placebo); none of these were associated with a change in vital signs [4]. Later analysis with 1035 patients showed an adverse event rate of 1.4% in the treatment group (versus 1.0% in the placebo group); however, hypoxia was not reported as an adverse event in this report [7].

The adverse event in our patient occurred within 3 minutes of infusion and consisted of hypoxia, swelling, petechial rash, and flushing. It is difficult to distinguish whether this represented a true allergic immunoglobulin E(IgE)-mediated, or anaphylactic, reaction as opposed to a non-IgE-mediated anaphylactoid reaction (also known as non-allergic anaphylaxis by the World Allergy Organization [9]) that can often result from direct activation of complement, mast cells, or basophils by non-immune mechanisms [10]. While the timing is concerning for a true anaphylactic reaction, the fact that he had never been exposed to either antibody suggests that this was in fact an anaphylactoid reaction, which often responds to steroids and antihistamines [11]. However, this does not necessarily exclude anaphylaxis, as he may have had prior exposure to ingredients in the infusion other than the antibodies themselves [12]. Of note, the patient did have known immunologic abnormalities, including splenomegaly and neutropenia, that may have predisposed him to a cytokine-mediated reaction. However, clinically, anaphylaxis and anaphylactoid reactions appear identical, and have both appeared in the context of COVID-directed therapies including vaccines [13]; given how rapidly anaphylaxis can progress, it is often safer to assume anaphylaxis and give epinephrine. As our patient had lost his intravenous line, we were fortunate that his reaction self-resolved with oral diphenhydramine alone. However, given the risk of aspiration with oral medications, intramuscular epinephrine would likely be a better choice in this circumstance.

The type of reaction has implications in whether or not to rechallenge; an anaphylactoid reaction may be more amenable to a rechallenge of the infusion at a slower rate with pre-medication, but a true anaphylactic reaction may preclude restarting the infusion [14]. As this was a very short hospital stay and the family was transferred from an outside hospital solely for the purpose of the infusion, and given that they did not wish to pursue further treatment, further testing such as a blood counts, liver function, basophil histamine release testing, or radioallergosorbent testing was not pursued. Unfortunately, the lack of laboratory data, such as IgE levels before and after infusion, does limit our ability to determine more precisely what happened.

At this time, it is difficult to draw generalized conclusions about what may or may not predispose a patient to such a reaction; however, the presence of skilled nursing and transfusion reaction medications at bedside was critical in stabilizing our patient. In particular, the nurse at bedside had performed similar infusions with bamlanivimab and etesevimab with previously approved patients and was able to quickly identify the adverse event. Given the novelty of this treatment in children, prior experience in administration is highly recommended to protect against poor outcomes, as is easy availability of epinephrine and tools for airway management.

## Conclusion

Though the benefits of bamlanivimab and etesevimab are compelling based on previous clinical trials, there is a possibility of an infusion reaction in children, which may be either anaphylactic or anaphylactoid. Communication and preparedness regarding this possibility, including having nurses experienced with infusion and having epinephrine and tools for airway management on-hand, will help ensure maximal benefit of this treatment with minimal risk.

## Data Availability

Data sharing is not applicable to this article as no datasets were generated or analyzed during the current study.

## Abbreviations

FDA: Food and Drug Administration
EUA: Emergency use authorization
RT-PCR: Reverse-transcription polymerase chain reaction
